# Neighborhood Food Environment and Walkability Predict Obesity in New York City

**DOI:** 10.1289/ehp.11590

**Published:** 2008-10-02

**Authors:** Andrew Rundle, Kathryn M. Neckerman, Lance Freeman, Gina S. Lovasi, Marnie Purciel, James Quinn, Catherine Richards, Neelanjan Sircar, Christopher Weiss

**Affiliations:** 1 Department of Epidemiology, Mailman School of Public Health; 2 Institute for Social and Economic Research and Policy; 3 Urban Planning, Graduate School of Architecture, Planning, and Preservation and; 4 Robert Wood Johnson Foundation Health and Society Scholars Program, Columbia University, New York, New York, USA

**Keywords:** neighborhood studies, obesity, retail food environment, walkability

## Abstract

**Background:**

Differences in the neighborhood food environment may contribute to disparities in obesity.

**Objectives:**

The purpose of this study was to examine the association of neighborhood food environments with body mass index (BMI) and obesity after control for neighborhood walkability.

**Methods:**

This study employed a cross-sectional, multilevel analysis of BMI and obesity among 13,102 adult residents of New York City. We constructed measures of the food environment and walkability for the neighborhood, defined as a half-mile buffer around the study subject’s home address.

**Results:**

Density of BMI-healthy food outlets (supermarkets, fruit and vegetable markets, and natural food stores) was inversely associated with BMI. Mean adjusted BMI was similar in the first two quintiles of healthy food density (0 and 1.13 stores/km^2^, respectively), but declined across the three higher quintiles and was 0.80 units lower [95% confidence interval (CI), 0.27–1.32] in the fifth quintile (10.98 stores/km^2^) than in the first. The prevalence ratio for obesity comparing the fifth quintile of healthy food density with the lowest two quintiles combined was 0.87 (95% CI, 0.78–0.97). These associations remained after control for two neighborhood walkability measures, population density and land-use mix. The prevalence ratio for obesity for the fourth versus first quartile of population density was 0.84 (95% CI, 0.73–0.96) and for land-use mix was 0.91 (95% CI, 0.86–0.97). Increasing density of food outlets categorized as BMI-unhealthy was not significantly associated with BMI or obesity.

**Conclusions:**

Access to BMI-healthy food stores is associated with lower BMI and lower prevalence of obesity.

The United States faces an epidemic of overweight and obesity (Ogden et al. 2006). Analyses of National Health and Nutrition Examination Survey data for 1999–2004 show that 32% of Americans > 20 years of age are obese (Ogden et al. 2006). New York City Department of Health and Mental Hygiene statistics show that New York City, our study site, likewise faces high rates of overweight/ obesity (Roberts et al. 2005). There is a growing understanding that the availability of residential neighborhood resources that support physical activity and healthy food choices may influence obesity rates (Larkin 2003; Rao et al. 2007).

Previous studies linking food environment measures with dietary intake or obesity have found mixed results. Proximity to supermarkets has been positively associated with consumption of a healthy diet (Laraia et al. 2004; Morland et al. 2002; Zenk et al. 2005) and negatively associated with overweight or obesity (Morland et al. 2006). Individuals with access to lower-priced fruits and vegetables have a lower body mass index (BMI) (Sturm and Datar 2005), whereas those living near convenience stores have higher rates of overweight and obesity (Morland et al. 2006). To date, however, there is no evidence that proximity to fast-food restaurants influences diet or obesity risk (Burdette and Whitaker 2004; Jeffery et al. 2006; Liu et al. 2007). Most analyses relating the density of other types of restaurants or grocery stores to BMI or obesity risk found no significant association (Jeffery et al. 2006; Liu et al. 2007; Powell et al. 2007; Sturm and Datar 2005).

Two concerns can be raised about this existing literature. First, most analyses do not control for built environment characteristics, such as land-use mix and population density, associated with pedestrian travel and lower BMI, which also tend to covary with density of retail food outlets (Rundle et al. 2007). Second, with a few exceptions (e.g., Morland et al. 2006), most studies relating the food environment to diet or body weight focus on just a few types of food outlets rather than considering the food environment as a whole. Because density measures for different types of food outlets are likely to be correlated with each other and with commercial space availability in general, their individual associations with BMI may be difficult to disentangle.

In this study we related the food environment to BMI and obesity in New York City. Addressing the concerns noted above, the analysis controls for neighborhood and built environment features already shown to influence BMI and includes measures of all restaurants, grocery stores, and specialty food vendors in the city (Rundle et al. 2007). To address problems of multicollinearity raised by simultaneous inclusion of a large number of food outlet measures, we constructed density measures for three food environment categories: BMI-healthy food outlets such as supermarkets and fruit and vegetable markets, BMI-unhealthy food outlets such as fast-food restaurants and convenience stores, and a BMI-intermediate category.

## Materials and Methods

The analyses presented here employed data collected during the baseline enrollment of subjects for the New York Cancer Project, a study of residents of New York City and the surrounding suburbs that has been described extensively elsewhere (Mitchell et al. 2004; Rundle et al. 2007). Of the total sample, 14,147 individuals had geocoded addresses falling within New York City boundaries, and 13,102 had a BMI < 70 and complete data for objectively measured height and weight and questionnaire measures of age, race and ethnicity, sex, income, and educational attainment. Table 1 shows descriptive statistics for individual characteristics. The demographic profile and spatial distribution of the sample are similar to those derived from the 2000 U.S. Census and from the 2002 New York City Community Health Survey (Rundle et al. 2007). Analyses of BMI, individual demographic variables, and appended neighborhood characteristics were approved by the Columbia University Medical Center Institutional Review Board.

### Neighborhood measures

We defined a study subject’s neighborhood as a half-mile (805 m) “network buffer” around his or her residential address, comprising locations reachable within a half-mile walk along the street network. Most urban planners assume that a half-mile is a walkable distance (Agrawal et al. 2008; Calthorpe 1993; Cervero 2006). We constructed sociodemographic and built environment measures, including food environment variables, for each individual’s neighborhood. To control for the effects of neighborhood composition on BMI, our models adjusted for the proportion of residents below the federal poverty line, proportion black, and proportion Hispanic using data from the 2000 U.S. Census summary file 3 (U.S. Census Bureau 2000).

We assessed the possible confounding effects of the following measures of neighborhood walkability: population density, density of bus and subway stops, percentage of commuters using public transit, land-use mix, and proportion of land zoned to permit commercial development (Rundle et al. 2007). We calculated population density, expressed as persons per square kilometer of land area, and the percentage of commuters using public transit from 2000 U.S. Census data (U.S. Census Bureau 2000). We based the numbers of bus and subway stops per square kilometer on data from the Department of City Planning (DCP). We constructed the proportion of the buffer zoned to permit commercial development and a measure of residential/commercial land-use mix using the Primary Land Use Tax Lot Output data, a parcel-level data set also available from DCP. Land-use mix is an index of the extent to which a neighborhood supports both commercial and residential lands uses, with the index tending toward 1 as the mix of residential and commercial floor area approaches a 1:1 ratio.

### Food environment measures

We derived food environment measures from 2001 data purchased from Dun & Bradstreet (D&B; unpublished data). The data include business name, geocoded location, and detailed Standard Industrial Classification (SIC) industry codes (http://www.osha.gov/pls/imis/sic_manual.html) for food establishments. *A priori*, we grouped food outlets into categories hypothesized to provide BMI-healthy or BMI-unhealthy food, with one intermediate category for food outlets whose classification was uncertain. We classified supermarkets and fruit and vegetable markets as BMI-healthy based on evidence associating proximity to supermarkets with better dietary patterns and lower BMI (Laraia et al. 2004; Morland et al. 2002, 2006; Zenk et al. 2005), lower fruit and vegetable prices with slower growth in BMI (Powell et al. 2007; Sturm and Datar 2005), and daily vegetable consumption with lower rates of obesity (Lahti-Koski et al. 2002). Although supermarkets sell a range of food, including both healthy and unhealthy options, we consider them healthy food outlets because they offer local residents the opportunity to purchase healthy food. No evidence is available linking natural food stores to diet or BMI, but food products typically available at natural food stores tend to be healthier; thus, we also categorized natural food stores as BMI-healthy food outlets.

The category of BMI-unhealthy food outlets included fast-food restaurants, a choice based on extensive evidence linking fast-food consumption with high energy intake, fat intake, BMI, and weight gain (Befort et al. 2006; Bowman and Vinyard 2004; Duerksen et al. 2007; Duffey et al. 2007; French et al. 2001; Jeffery et al. 2006; Jeffery and French 1998; Thompson et al. 2004). The BMI-unhealthy food index also included convenience stores (Morland et al. 2006) and meat markets (Gillis and Bar-Or 2003; Lahti-Koski et al. 2002). We classified pizzerias, bakeries, and candy and nut stores as BMI-unhealthy based on the energy density of the types of foods sold there. Because “bodegas” or very small grocery stores tend to sell energy-dense foods and few fruits and vegetables, they were classed as BMI-unhealthy (Kaufman and Karpati 2007).

The BMI-intermediate category comprised food outlets for which evidence was insufficient for placement in the other two categories. This category included non-fast-food restaurants—that is, restaurants excluding fast food and pizzerias. Although eating food prepared away from home has sometimes been associated with poor diet and higher weight (Gillis and Bar-Or 2003; Guthrie et al. 2002; Yao et al. 2003), research on consumption of food from non-fast-food restaurants has found no effect on weight or weight gain (Duffey et al. 2007; Jeffery et al. 2006; Thompson et al. 2004), and one study found higher vegetable consumption among adolescents who ate more frequently at non-fast-food restaurants (Befort et al. 2006). The intermediate category also includes medium-sized grocery stores and specialty stores, as well as fish markets. Although some evidence associates fish intake with weight loss (Thorsdottir et al. 2007), fish markets in New York City often sell fried fish and fried seafood for immediate consumption; thus, the implication of this food outlet type for weight is unclear.

We identified most food outlet types by SIC code number alone: fruit and vegetable markets (#5431), natural or health food stores (#549901), fish markets (#542101), specialty food stores (#5451 and #5499, excluding #549901), convenience stores (#541102), bakeries (#5461), candy and nut stores (#5441), and meat markets (#542102). We distinguished three categories of grocery stores, excluding convenience stores. We identified “supermarkets” as grocery stores (#5411) with at least $2 million in annual sales or, for establishments with missing data on annual sales, at least 18 employees. (Among establishments with annual sales data, 18 employees was the threshold at which at least half had annual sales of ≥ $2 million.) “Mediumsized grocery stores” were nonsupermarket groceries with at least five employees. “Bodegas” were grocery stores with fewer than five employees. We identified national-chain fast-food restaurants through text searches in the D&B “company name” and “tradestyle” fields for names appearing in Technomic Inc.’s list of the top 100 limited-service chain brands (Technomic Inc. 2006). We identified as local fast food those restaurants that were not already identified as a national-chain fast-food restaurant and that had an SIC code indicating fast food (#58120300, #58120307, or #58120308), as well as the restaurants with names matching those on this list of local fast-food restaurants. We identified non-fast-food restaurants with “pizza” or “pizzeria” in their name, or with SIC codes of #58120600, #58120601, or #58120602, as pizzerias. We categorized all other establishments with an SIC code of 5812 as non-fast-food restaurants.

The density per square kilometer of establishments falling within each of these three categories was calculated for each subject’s unique network buffer. Subjects were then categorized into increasing quintiles for each of the three food outlet categories.

### Statistical analysis

We calculated adjusted mean BMI for each quintile of retail density for the three food categories using cross-sectional, multilevel modeling (Diez Roux 2000) with the Proc Mixed procedure (Singer 1998) in SAS (version 9; SAS Institute Inc., Cary, NC). Because each of the neighborhood-level measures was generated for each individual’s address, we treated the neighborhood variables as level 1 variables. We expected intercor-relations among individuals, reflecting similarity among those living in proximity to each other, to exist across a geographic scale larger than the half-mile buffers. To account for this, we estimated our multilevel models with community district as a level 2 clustering factor. New York City’s 59 community districts correspond to named areas such as the Upper West Side and Chinatown. Although we measured no predictive variables at level 2, the use of this nested data structure allowed for valid estimation of standard errors. We adjusted analyses for individual and neighborhood sociodemographic characteristics and then for the five neighborhood walkability measures. We evaluated the five walkability measures as possible confounders individually and in combination. We mutually adjusted all analyses for quintiles of each of the three food categories.

We calculated separate prevalence ratios for overweight and obesity compared with normal weight for increasing quintiles of retail food density categories using Poisson regression with robust variance estimates (Spiegelman and Hertzmark 2005). We used community district as a clustering variable to correct the standard errors for intercorrelations among individuals across larger areas of the city and to generate robust SE estimates.

## Results

The data set initially received from D&B included 32,949 retail food businesses for New York City. After correction of geocoded addresses and removal of duplicate records, businesses likely to be defunct, and records likely to represent back offices and corporate offices, the data set included 29,976 businesses, of which 29,858 fell within the bounds of study subjects’ neighborhoods. Table 2 displays descriptive statistics for the BMI-healthy, BMI-unhealthy, and BMI-intermediate categories as well as for specific food outlet types. Density of intermediate and unhealthy food outlets was much higher than density of healthy food outlets. Almost all study subjects lived within a half-mile of an unhealthy food outlet, with an average density of 31 such outlets per square kilometer. By contrast, only 82% lived within a half-mile of a healthy food outlet, with an average density of four outlets per square kilometer. Density measures for food outlet types were significantly correlated across neighborhoods, with correlation coefficients ranging from 0.38 (convenience stores and supermarkets) to 0.85 (non-fast-food restaurants and pizza restaurants).

**Figure 1:**
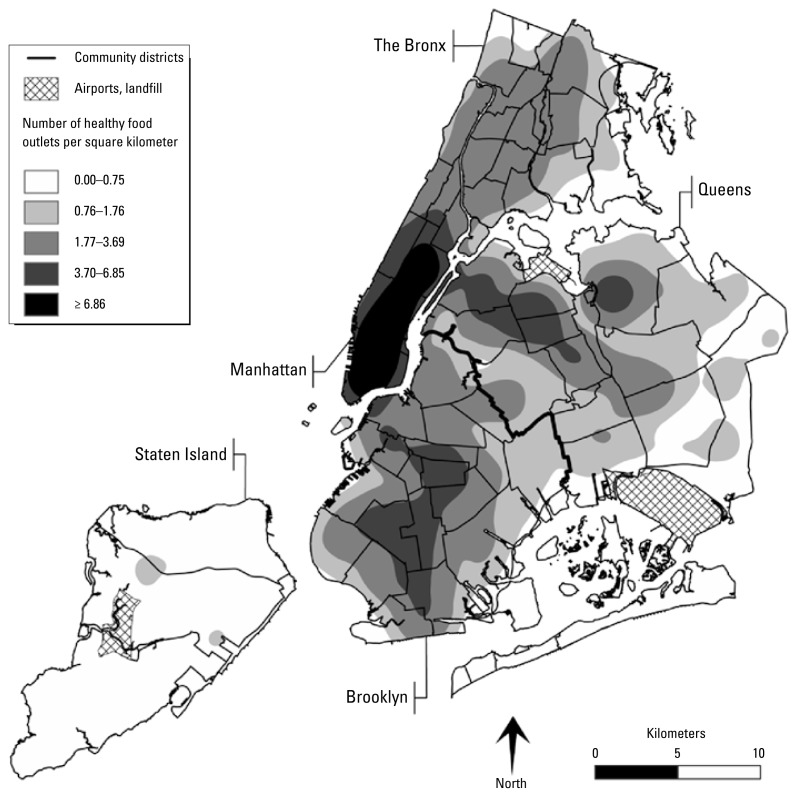
Density of BMI-healthy food outlets in New York City: Kernel Density Estimation (KDE) map illustrating the density of BMI-healthy food outlets. This KDE continuous surface was created with ArcGIS Spatial Analyst (ESRI, Redlands, CA), which uses a distance decay quadratic kernel function. Input processing parameters included a half-mile bandwidth and 1,545 discrete points representing the locations of supermarkets, fruit and vegetable markets, and natural food stores.

Figure 1 maps the density of BMI-healthy food outlets, expressed in outlets per square kilometer, across the city. Outlet density was highest in high-walkability areas of the city, such as Manhattan, and lowest in low-walkability areas, such as Staten Island. Outlet density also varied by neighborhood income and race/ethnic composition, with higher densities in affluent and predominantly white neighborhoods in the southern half of Manhattan and lower densities in the poor and predominantly black or Latino neighborhoods in the northern half of Manhattan and in the South Bronx. To reduce the risk of confounding, the multivariate analyses controlled statistically for individual-level race/ethnicity and education and neighborhood-level poverty rate and race/ethnic composition, as well as indices of neighborhood walkability, including population density and land-use mix.

**Figure 2:**
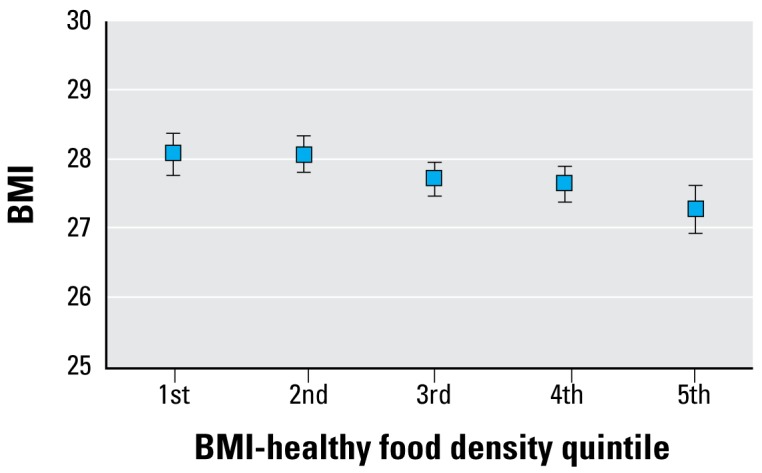
Adjusted mean BMI (± 95% CI) by BMI-healthy food density quintiles. Analysis is adjusted for the density of BMI-intermediate and BMI-unhealthy food outlets and for age, sex, race/ ethnicity, education, neighborhood sociodemographic characteristics, and population density.

Multilevel analyses of the association between BMI and the food environment measures showed significant associations only with access to BMI-healthy food. We also assessed possible confounding effects of built environment variables. Population density, which has previously been inversely associated with BMI in analyses of the same data set, had an appreciable confounding effect, but further control for land-use mix, percent commercial area, and access to and neighborhood use of public transit did not alter the results. Table 3 shows adjusted mean BMI for each quintile of the three food categories and the median density of food outlets for each category; Figure 2 displays the association between healthy food outlet density and BMI based on this analysis. The adjusted mean BMI in the fifth quintile of healthy food was 0.80 units [95% confidence interval (CI), 0.27–1.32, *p* < 0.01] lower than in the first quintile of healthy food. Population density and land-use mix remained significantly inversely associated with BMI after controlling for measures of the food environment. Increasing density of the BMI-unhealthy and BMI-intermediate food categories was not associated with BMI, and analyses of selected subcategories of BMI-unhealthy food (fast food, pizzerias, and convenience stores) found no significant associations.

Because there was little difference in the adjusted mean BMI of individuals living in the first and second quintile of BMI-healthy food density, we collapsed these two categories into a single reference category to increase statistical power for analyses of the prevalence of overweight and obesity. The reference category had a median density of 0.76 healthy food outlets per square kilometer. Table 4 shows the prevalence ratios for overweight and obesity by increasing density of healthy food outlets, increasing population density, and land-use mix. Controlling for population density and land-use mix, the prevalence of overweight and obesity were both lower among individuals with the highest density of healthy food outlets. Controlling for other features of the built environment did not alter the prevalence ratio for healthy food density.

Our previous work showed that increasing land-use mix and population density were inversely associated with BMI; this association remained after control for the density of BMI-healthy, BMI-unhealthy, and BMI-intermediate food outlets (Rundle et al. 2007). The prevalence ratio for obesity comparing the fourth and first quartiles of land-use mix was 0.91 (95% CI, 0.86–0.97) and comparing the fourth and first quartiles of population density was 0.84 (95% CI, 0.73–0.96).

## Discussion

The results presented here indicate that the food environment is significantly associated with body size net of individual and neighborhood characteristics and neighborhood walkability features. A higher local density of BMI-healthy food outlets was associated with a lower mean BMI, a lower prevalence of overweight, and a lower prevalence of obesity. BMI-unhealthy food stores and restaurants were far more abundant than healthy ones, but the density of these unhealthy food outlets was not significantly associated with BMI or with body size categories. Of studies relating the food environment to body size, this work is among the first to measure the food environment comprehensively and to account for the effects of other built environment factors associated with obesity. The apparent effect of the food environment, while modest, is net of the significant associations between indices of neighborhood walkability and BMI. Our prior work showed that built environment features related to walkability were associated with approximately a 10% difference in the prevalence of obesity (Rundle et al. 2007). Even after control for measures of the food environment, the estimated effects of these built environment variables remained and were of a similar magnitude. Considered together, food environment and neighborhood walkability may have a substantial effect on body size.

Although the observed associations between BMI and the density of BMI-healthy food establishments were consistent with expectations, we had also hypothesized that increasing density of BMI-unhealthy food options would be positively associated with BMI. Because the density of unhealthy food outlets is correlated with commercial activity in general as well as other features of the urban landscape that promote pedestrian activity, we expected that associations between unhealthy food density and BMI had been masked in prior research and would be observed after control for such built environment features. Consistent with other studies in this area, however, we found no association between density of unhealthy food and BMI or obesity. This lack of association may reflect the ubiquity of unhealthy food in an urban environment; as Table 2 shows, virtually all New York City neighborhoods provide many opportunities to eat poorly. In addition, unhealthy convenience foods may be consumed near the workplace or during travel about the city, making the density of unhealthy foods in the residential neighborhood less relevant. Alternatively, the null findings may reflect undercounting of unhealthy food outlets in the most disadvantaged urban neighborhoods. As the case of New York City shows, the penetration of national-chain fast food is low in some of the poorest neighborhoods; this niche in the food environment is filled by inexpensive ethnic restaurants selling high-calorie take-out food (Graham et al. 2006). Better measures of the food environment may show an association of unhealthy food outlets with body size.

One limitation of this study and, indeed, of most studies on this topic is that our data are observational and cross-sectional. Observed associations may be attributable to self-selection of individuals into neighborhoods that support their preferred lifestyle; for instance, individuals who prefer to consume healthy foods may move to neighborhoods with more healthy food outlets. Conversely, retailers selling healthy foods may choose to locate in neighborhoods where they believe the population will be most receptive to their products. In addition, questions might be raised about two potential sources of error in the food environment measures. The first is incomplete coverage of the D&B data. Because the D&B data are used primarily for marketing purposes, coverage may be less complete in areas less attractive to marketers, such as low-income neighborhoods. For error in the D&B database to bias our results, it would have to be correlated with the spatial distribution of BMI. Our analyses control for neighborhood sociodemographic composition, which may be an important correlate of measurement error in the D&B data. Second, measurement error may be caused by misclassification of food outlets into the BMI-healthy, BMI-unhealthy, and BMI-intermediate categories. Some food outlets, such as fruit and vegetable markets, are internally relatively homogeneous, whereas others, such as grocery stores or non-fast-food restaurants, may have significant internal heterogeneity. Within-category heterogeneity in food selection may bias food environment coefficients toward zero or create interactions between neighborhood composition and food environment characteristics. Although the analyses controlled for neighborhood sociodemographic composition and for land-use mix and commercial space, variables that might be expected to influence the extent of within-category measurement error, measurement error remains a concern. A further limitation is the mismatch between the time period of the survey (2000–2002) and the time period of food environment measures (2001), population census measures (2000), and land-use and zoning data (2003); because neighborhood demographic and built environment characteristics typically change slowly, these discrepancies should not affect the results significantly. Limitations also include the lack of an audit to verify types of food sold in different types of stores.

A distinctive feature of this study is its use of broad categories to characterize the food environment based on the existing literature. Although this analytic strategy sacrifices the opportunity to identify associations between specific food outlet types and BMI, it has several advantages. First, although some in the public health and medical communities, as well as the popular media, have focused on the contribution of fast food to the obesity epidemic, other types of food outlets also sell high-energy-density food; comprehensive measures of the food environment provide a more accurate account of the food choices available to urban residents (Stender et al. 2007; Wallis 2004). Second, density measures for the 14 individual food outlet categories are significantly correlated; reflecting this multi-collinearity, models including all 14 measures are quite unstable. Third, reducing the number of food outlet measures made it less likely that one would be significant simply by chance. Specific choices about how to group food outlet types can certainly be debated and can be tested in replication.

The research reported here adds to our knowledge about the relationship between the food environment and obesity with evidence that access to BMI-healthy food outlets such as supermarkets, fruit and vegetable markets, and natural food stores is inversely associated with obesity. This protective association is net of urban design features that promote pedestrian activity and lower BMI, as well as the density of other types of food outlets. Although not identifying a specific culprit within the retail food environment for the obesity epidemic, these analyses indicate that retail outlets providing opportunities for healthier food purchases are associated with lower BMI. If the results of our observational research are confirmed by future studies that permit causal inference, this evidence would suggest that increasing access to healthy food outlets is likely to do more to address the obesity epidemic than limiting unhealthy food outlets. Given the recent proliferation of initiatives to promote access to supermarkets, farmers markets, and fruit and vegetable stands and to limit fast-food outlets (Abdollah 2007; Lee 2007; Marter 2007), study of the causal relationship between the food environment and diet or body size should be a priority for future research.

## Figures and Tables

**Table 1:** Characteristics of the study population, New York City, 2000–2002 (*n* = 13,102).

Characteristic	Study population
Continuous variables [mean ± SD (median)]	
Age (years)	46.21 ± 10.55 (45.00)
BMI	27.73 ± 5.78 (26.60)
Categorical variables (%)	
Sex	
Men	36
Women	64
Race/ethnicity	
Asian	12
Black: African American	14
Black: Caribbean	5
Caucasian	47
Hispanic	20
Other	2
Educational attainment	
Some high school or less	13
High school graduate	22
Vocational school	2
Some college	21
College graduate	24
Graduate school	18
Body size	
Underweight	1
Normal weight	34
Overweight	37
Obese	28

**Table 2:** Descriptive statistics for food outlet density (stores/km^2^).

Food outlet type	Density (mean ± SD) (10th, 90th percentiles)	Percent buffers with this type of outlet
BMI-healthy		
Supermarkets	1.39 ± 1.77 (0, 3.89)	63.2
Fruit and vegetable stores	1.57 ± 2.11 (0, 4.03)	61.2
Natural/health food stores	1.31 ± 2.00 (0, 3.82)	54.3
Total	4.27 ± 4.95 (0, 10.98)	82.4
BMI-intermediate		
Other (non-fast-food) restaurants	38.85 ± 59.50 (4.16, 88.93)	98.9
Mediumsized grocery stores	2.10 ± 2.48 (0, 5.44)	71.4
Fish markets	0.83 ± 1.29 (0, 2.30)	78.6
Specialty food stores	1.66 ± 3.75 (0, 4.27)	56.8
Total	43.44 ± 65.15 (4.79, 99.21)	98.9
BMI-unhealthy		
Fast-food restaurants	3.44 ± 4.43 (0, 8.36)	72.5
Pizza restaurants	4.22 ± 4.27 (0.68, 9.56)	90.2
Convenience stores	1.42 ± 1.53 (0, 3.71)	67.1
Bodegas	15.16 ± 13.32 (1.59, 31.86)	95.5
Bakeries	3.61 ± 4.48 (0, 8.45)	83.3
Candy and nut stores	1.39 ± 2.10 (0, 3.72)	57.7
Meat markets	1.57 ± 1.99 (0, 3.93)	66.7
Total	30.81 ± 27.22 (4.88, 64.12)	98.8

**Table 3:** Adjusted mean BMI by food density quintiles.

Food density category	1st Quintile	2nd Quintile	3rd Quintile	4th Quintile	5th Quintile
BMI-healthy					
Median density (stores/km^2^)	0.00	1.13	2.62	4.95	10.98
Adjusted mean BMI^a^	28.06	28.05	27.70	27.63	27.26
95% CI	27.75–28.36	27.79–28.32	27.45–27.94	27.37–27.88	26.91–27.61
*p*-Value^b^		0.99	0.06	0.05	0.003
BMI-intermediate					
Median density (stores/km^2^)	4.79	12.23	23.18	37.44	99.26
Adjusted mean^a^	27.76	27.88	28.00	27.75	27.30
95% CI	27.34–28.18	27.57–28.19	27.72–28.27	27.45–28.06	26.87–27.73
*p*-Value^b^		0.57	0.37	0.98	0.22
BMI-unhealthy					
Median density (stores/km^2^)	4.88	12.50	24.94	38.41	64.19
Adjusted mean^a^	27.73	27.69	27.54	27.83	27.91
95% CI	27.30–28.16	27.36–28.01	27.28–27.81	27.52–28.14	27.48–28.34
*p*-Value^b^		0.83	0.49	0.75	0.64

a Adjusted for age, sex, race/ethnicity, education, neighborhood sociodemographic characteristics, and population density. Results for each food outlet category were also mutually adjusted for the other two food outlet categories.

b *p*-Value for difference in BMI comparing each quintile to the first quintile.

**Table 4:** Prevalence ratios (95% CIs) for overweight and obesity by increasing density of BMI-healthy food and indices of increasing neighborhood walkability.

Category	Normal versus overweight	Normal versus obese
Healthy food (quintiles)		
1–2	1	1
3	0.98 (0.92–1.05)	0.97 (0.91–1.03)
4	0.98 (0.92–1.05)	0.95 (0.89–1.02)
5	0.94 (0.88–1.01)	0.87 (0.78–0.97)
Population density (quartiles)		
1	1	1
2	0.96 (0.89–1.03)	0.94 (0.87–1.01)
3	0.91 (0.83–1.01)	0.89 (0.79–1.01)
4	0.84 (0.75–0.95)	0.84 (0.73–0.96)
Land-use mix (quartiles)		
1	1	1
2	1.00 (0.95–1.05)	0.99 (0.92–1.05)
3	0.94 (0.89–0.99)	0.98 (0.92–1.05)
4	0.92 (0.87–0.97)	0.91 (0.86–0.97)

Results were mutually adjusted and further adjusted for age, sex, race/ethnicity, education, neighborhood sociodemographics, and quintiles of unhealthy food density and intermediate food density.

## References

[b1-ehp-117-442] Abdollah T (2007). A strict order for fast food. Los Angeles Times (Los Angeles, CA).

[b2-ehp-117-442] Agrawal AW, Schlossberg M, Irvin K (2008). How far, by which route and why? A spatial analysis of pedestrian preference. Environ Behav.

[b3-ehp-117-442] Befort C, Kaur H, Nollen N, Sullivan DK, Nazir N, Choi WS (2006). Fruit, vegetable, and fat intake among non-Hispanic black and non-Hispanic white adolescents: associations with home availability and food consumption settings. J Am Diet Assoc.

[b4-ehp-117-442] Bowman SA, Vinyard BT (2004). Fast food consumption of US adults: impact on energy and nutrient intakes and overweight status. J Am Coll Nutr.

[b5-ehp-117-442] Burdette HL, Whitaker RC (2004). Neighborhood playgrounds, fast food restaurants, and crime: relationships to overweight in low-income preschool children. Prev Med.

[b6-ehp-117-442] Calthorpe P (1993). The Next American Metropolis: Ecology, Community, and the American Dream.

[b7-ehp-117-442] Cervero R (2006). Alternative approaches to modeling the travel-demand impacts of Smart Growth. J Am Plann Assoc.

[b8-ehp-117-442] Diez Roux AV (2000). Multilevel analysis in public health research. Annu Rev Public Health.

[b9-ehp-117-442] Duerksen SC, Elder JP, Arredondo EM, Ayala GX, Slymen DJ, Campbell NR (2007). Family restaurant choices are associated with child and adult overweight status in Mexican-American families. J Am Diet Assoc.

[b10-ehp-117-442] Duffey KJ, Gordon-Larsen P, Jacobs DR, Williams OD, Popkin BM (2007). Differential associations of fast food and restaurant food consumption with 3-y change in body mass index: the Coronary Artery Risk Development in Young Adults study. Am J Clin Nutr.

[b11-ehp-117-442] French SA, Story M, Neumark-Sztainer D, Fulkerson JA, Hannan P (2001). Fast food restaurant use among adolescents: associations with nutrient intake, food choices and behavioral and psychosocial variables. Int J Obes.

[b12-ehp-117-442] Gillis LJ, Bar-Or O (2003). Food away from home, sugar-sweetened drink consumption and juvenile obesity. J Am Coll Nutr.

[b13-ehp-117-442] Graham R, Kaufman L, Novoa Z, Karpati A (2006). Eating In, Eating Out, Eating Well: Access to Healthy Food in North and Central Brooklyn.

[b14-ehp-117-442] Guthrie JF, Lin BH, Frazao E (2002). Role of food prepared away from home in the American diet, 1977–78 versus 1994–96: changes and consequences. J Nutr Educ Behav.

[b15-ehp-117-442] Jeffery R, Baxter J, McGuire M, Linde J (2006). Are fast food restaurants an environmental risk factor for obesity?. Int J Behav Nutr Phys Act.

[b16-ehp-117-442] Jeffery RW, French SA (1998). Epidemic obesity in the United States: are fast foods and television viewing contributing?. Am J Public Health.

[b17-ehp-117-442] Kaufman L, Karpati A (2007). Understanding the sociocultural roots of childhood obesity: food practices among Latino families of Bushwick, Brooklyn. Soc Sci Med.

[b18-ehp-117-442] Lahti-Koski M, Pietinen P, Heliovaara M, Vartiainen E (2002). Associations of body mass index and obesity with physical activity, food choices, alcohol intake, and smoking in the 1982–1997 FINRISK studies. Am J Clin Nutr.

[b19-ehp-117-442] Laraia BA, Siega-Riz AM, Kaufman JS, Jones SJ (2004). Proximity of supermarkets is positively associated with diet quality index for pregnancy. Prev Med.

[b20-ehp-117-442] Larkin M (2003). Can cities be designed to fight obesity?. Lancet.

[b21-ehp-117-442] Lee J (2007). Aiming to put more carts and better food on the streets. New York Times (New York City).

[b22-ehp-117-442] Liu GC, Wilson JS, Qi R, Ying J (2007). Green neighborhoods, food retail and childhood overweight: differences by population density. Am J Health Promot.

[b23-ehp-117-442] Marter M (2007). Farmers markets go wild. Philadelphia Inquirer (Philadelphia).

[b24-ehp-117-442] Mitchell MK, Gregersen PK, Johnson S, Parsons R, Vlahov D (2004). The New York Cancer Project: rationale, organization, design, and baseline characteristics. J Urban Health.

[b25-ehp-117-442] Morland K, Diez Roux AV, Wing S (2006). Supermarkets, other food stores, and obesity—the atherosclerosis risk in communities study. Am J Prev Med.

[b26-ehp-117-442] Morland K, Wing S, Diez Roux AV (2002). The contextual effect of the local food environment on residents’ diets: the atherosclerosis risk in communities study. Am J Public Health.

[b27-ehp-117-442] Ogden CL, Carroll MD, Curtin LR, McDowell MA, Tabak CJ, Flegal KM (2006). Prevalence of overweight and obesity in the United States, 1999–2004. JAMA.

[b28-ehp-117-442] Powell LM, Slater S, Mirtcheva D, Bao YJ, Chaloupka FJ (2007). Food store availability and neighborhood characteristics in the United States. Prev Med.

[b29-ehp-117-442] Rao M, Prasad S, Adshead F, Tissera H (2007). The built environment and health. Lancet.

[b30-ehp-117-442] Roberts M, Kerker B, Mostashari F, Van Wye G, Thorpe LE (2005). Obesity and Health: Risks and Behaviors.

[b31-ehp-117-442] Rundle A, Diez Roux AV, Freeman LM, Miller D, Neckerman KM, Weiss CC (2007). The urban built environment and obesity in New York City: a multilevel analysis. Am J Health Promot.

[b32-ehp-117-442] Singer J (1998). Using SAS PROC MIXED to fit multilevel models, hierarchical models, and individual growth models. J Educ Behav Stat.

[b33-ehp-117-442] Spiegelman D, Hertzmark E (2005). Easy SAS calculations for risk or prevalence ratios and differences. Am J Epidemiol.

[b34-ehp-117-442] Stender S, Dyerberg J, Astrup A (2007). Fast food: unfriendly and unhealthy. Int J Obes.

[b35-ehp-117-442] Sturm R, Datar A (2005). Body mass index in elementary school children, metropolitan area food prices and food outlet density. Public Health.

[b36-ehp-117-442] Technomic Inc (2006). Sales and % Change for Top 100 Restaurants.

[b37-ehp-117-442] Thompson OM, Ballew C, Resnicow K, Must A, Bandini LG, Cyr H (2004). Food purchased away from home as a predictor of change in BMI Z-score among girls. Int J Obes.

[b38-ehp-117-442] Thorsdottir I, Tomasson H, Gunnarsdottir I, Gisladottir E, Kiely M, Parra MD (2007). Randomized trial of weight-loss-diets for young adults varying in fish and fish oil content. Int J Obes.

[b39-ehp-117-442] U.S. Census Bureau (2000). 2000 Census Summary File 3.

[b40-ehp-117-442] Wallis C (2004). The obesity warriors. Time Magazine.

[b41-ehp-117-442] Yao MJ, McCrory MA, Ma GS, Tucker KL, Gao SJ, Fuss P (2003). Relative influence of diet and physical activity on body composition in urban Chinese adults. Am J Clin Nutr.

[b42-ehp-117-442] Zenk SN, Schulz AJ, Hollis-Neely T, Campbell RT, Holmes N, Watkins G (2005). Fruit and vegetable intake in African Americans—income and store characteristics. Am J Prev Med.

